# Even a worm will turn: An atypical presentation of hydatid disease

**DOI:** 10.4102/sajid.v39i1.661

**Published:** 2024-09-09

**Authors:** Piero Saieva, Tamsin Lovelock, Thabiet Jardine

**Affiliations:** 1 Department of Medicine, Karl Bremer Hospital, Cape Town, South Africa; 2 Division of Infectious Diseases, Department of Internal Medicine, Faculty of Medicine and Health Sciences, Stellenbosch University and Tygerberg Hospital, Cape Town, South Africa; 3 Department of Internal Medicine, Faculty of Medicine and Health Sciences, Stellenbosch University and Tygerberg Hospital, Cape Town, South Africa

**Keywords:** hydatid disease, hydatid, cyst, cystic echinococcosis, *Echinococcus*

## Abstract

Hydatid disease (cystic echinococcosis) is a neglected zoonosis, often incidentally detected in its late stages. The clinical manifestations depend on the location and dimensions of the cysts, with the liver and lungs frequently affected. This case report describes thrombocytopenia, an unusual haematological complication of hydatid disease. We use this case to highlight the role that platelets play in various parasitic infections and to advocate for further research into the role of platelets in hydatid disease.

**Contribution:**

We draw attention to a less well-known complication of hydatid disease.

## Introduction

Hydatid disease is a neglected zoonosis caused by the *Echinococcus* genus of tapeworm. The World Health Organization estimates that 19 300 lives and 871 000 disability-adjusted life years are lost globally each year due to cystic echinococcosis.^1^ There is a dearth of data regarding its incidence in South Africa, but it is known to be more common among sheep-farming communities, especially in the Eastern Cape province.^2^ In the life cycle of cystic echinococcosis, infection is caused by ingesting the parasite in its larval stage. Domestic and wild canines are definitive hosts, and various mammals such as sheep and rodents are intermediate hosts. Humans may become accidental hosts by ingesting food, water or soil contaminated by faecal matter that contains *Echinococcus* oocytes from infected dogs.^2^ The initial phase of primary infections is always asymptomatic and may be protracted, with latent phases of over 50 years being reported.^2,3^ The clinical presentation of hydatid disease is usually related to local pressure effects, dependent on the sites and sizes of the cysts.^2^ However, in this case report, we describe an unusual systemic complication of hydatid disease leading to severe consequences for the patient. ‘Even a worm may turn’ is a well-known line from William Shakespeare’s play *Henry VI, Part 3*, expressing that even a worm will revolt at some stage when pushed too far, much like the *Echinococcus* tapeworm in this case.

## Case presentation

**FIGURE 1:**
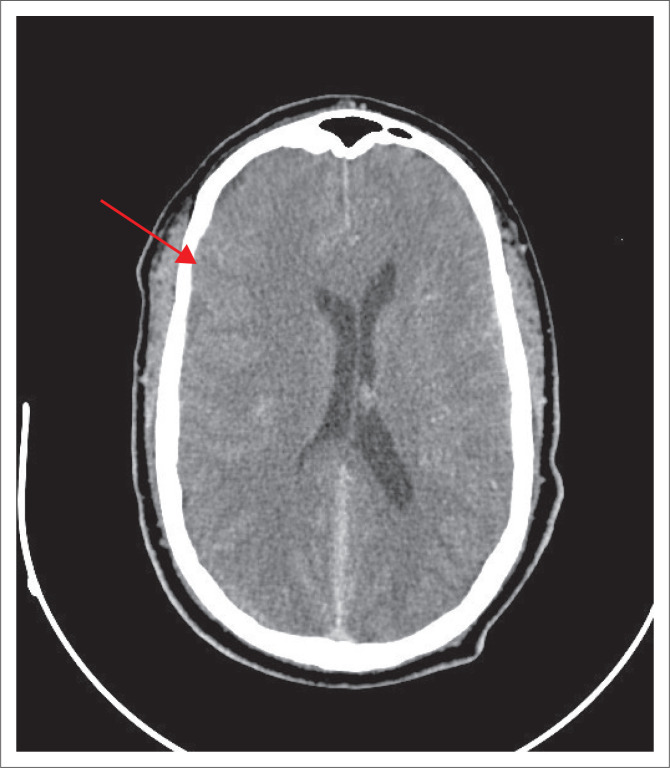
Computed tomography of the brain showing midline shift of 4 mm with evidence of a right subdural haemorrhage.

A 63-year-old man, originally from the Eastern Cape province of South Africa, but residing in the Western Cape for the last 5 years, presented to the emergency centre at a district hospital in Cape Town with new-onset generalised tonic-clonic seizures. Vital signs were normal with neither meningism nor focal neurological signs on examination. His initial Glasgow Coma Scale score was 14/15. His medical history included hypertension, obesity and type 2 diabetes mellitus. A routine chest radiograph (unfortunately of poor technical quality) showed a left lower lobe opacification. Blood results showed a bicytopenia with a haemoglobin count of 8.0 g/dL and a platelet count of 54 × 109/L. The white cell count was 8.05 × 109/L. His differential count showed a normal eosinophil count (0.15 × 109/L) as well as normal neutrophil, lymphocyte and monocyte counts. Urea, creatinine and electrolyte levels were normal, with HIV ELISA and treponemal antibody tests being negative. Computed tomography of the brain (CTB, Figure 1) showed a right subdural haemorrhage with 4 mm midline shift and early right uncal herniation.

The patient was discussed with the neurosurgery consultant of the tertiary referral hospital who advised that no surgical intervention was required at that stage. The patient remained under neurological observation, under the care of the general surgery team of the district hospital where dexamethasone to reduce tissue oedema and phenytoin to prevent further seizures were initiated. No further seizures were observed, and the patient returned to his neurological baseline within 24 h post admission. As a repeat CTB performed 3 days later was unchanged; plans for discharge were made. Prior to discharge, the surgical team noted the bicytopenia (normocytic anaemia and thrombocytopenia) on his initial blood results, and an internal medicine consultation was therefore requested.

**FIGURE 2:**
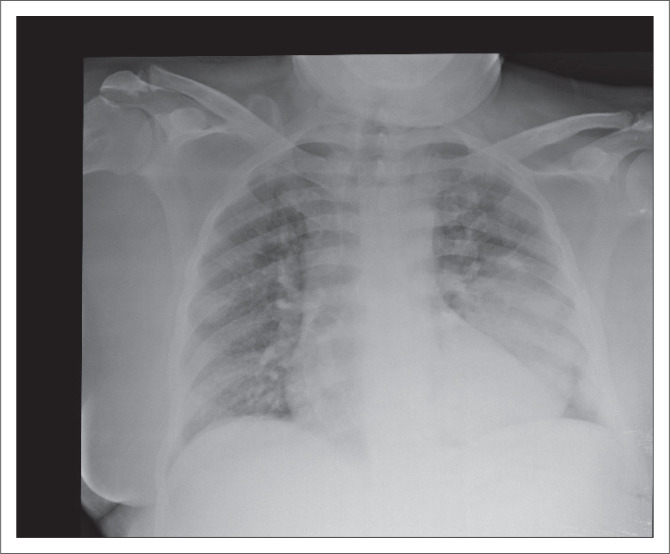
Chest radiograph showing a dense opacification in left lower lobe.

**FIGURE 3:**
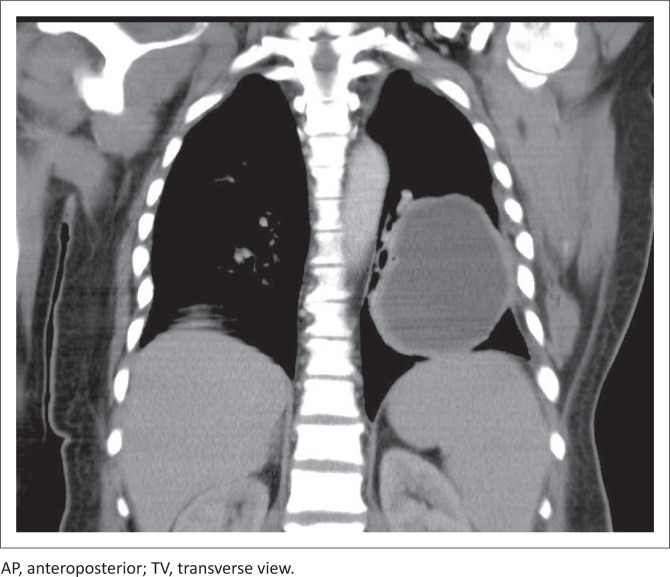
Computed tomography scan of the chest showing cystic lesion measuring 91 mm AP × 90 mm TV in left lower lobe.

On further history taking, no possible causative drugs were identified. Clinically, there were no petechiae, purpura or ecchymoses. There was no clinically appreciable lymphadenopathy or hepatosplenomegaly. Laboratory investigations revealed no evidence for nutritional deficiency, haemolysis, liver dysfunction and thyroid disease to account for the bicytopenia. An antinuclear antibody test and rheumatoid factor were also negative. A repeat chest radiograph (Figure 2) was requested (due to the poor quality of his initial radiograph), which revealed a well-circumscribed opacification in the left lower lobe. A computed tomography (CT) scan of the chest and abdomen (Figure 3) showed a cystic lesion with enhancing capsule due to calcifications in the left lower lobe measuring 91 mm anteroposterior × 90 mm transverse. No cysts were identified in the abdomen. While the differential for a lung mass is quite broad, given the typical CT findings, a presumptive diagnosis of hydatid disease was made. Treatment with albendazole 400 mg 12 hourly, with a fatty meal, was initiated. The pulmonology unit was consulted at the tertiary referral hospital for possible surgical intervention, given the large size of the cyst. The advice was to continue albendazole until the patient could be seen for an ambulatory consultation in 3 months. The pulmonology team opted for medical management with ongoing albendazole without surgery which was also the patient’s preference. Serum *Echinococcus* ELISA was negative. It is well documented that serology may be negative in early disease or following calcification of the cyst.^3^ It is also documented that the sensitivity of the *Echinococcus* serology is around 50% – 60% in cystic hydatid disease of the lung although better than cystic hydatid disease of the brain.^4^ The patient’s platelet count began to rise within a few days. Notably, the patient had also received high-dose dexamethasone for a week. Further recovery in the platelet function was noted after continuing the albendazole and stopping dexamethasone as shown in Table 1, with his haemoglobin remaining around 7 g/dL.

**TABLE 1:** White cell count, haemoglobin concentration and platelet count.

Date	26 May 2023†	30 May 2023	05 June 2023‡	09 June 2023	18 September 2023	08 October 2023
White cell count (×10^9^/L)	8.00	7.36	7.00	4.41	4.80	7.00
Haemoglobin concentration (g/dL)	7.5	7.9	7.2	9.0	7.4	7.2
Platelet count (×10^9^/L)	55	Rejected	57	79	194	287

† , Dexamethasone treatment initiated between 26 May 2023 and 30 May 2023.

‡ , Dexamethasone treatment stopped and albendazole initiated on the 05 June 2023.

## Discussion

Platelets have previously been thought to play a minor role in the immune system; however, their contribution to the immune system is receiving more attention.^5^ Platelets play an important role in both innate and adaptive immunity.^5^ Their roles include intervention against microbial threats, recruitment and promotion of innate effector cell functions, modulating antigen presentation and enhancing adaptive immune responses. The role that platelets play in relation to bacteria has been well established, but platelet function (or sometimes lack of function) related to various parasites still requires investigation.^6,7,8^ Recent research has shed some light on the various impacts that platelets have on some parasites and the impact of some parasites on platelets.

Platelets are well known for their contribution to the body’s response to the *Plasmodium* species, with thrombocytopenia being described even in completely asymptomatic *Plasmodium* infections.^9^ This is thought to relate to the various platelet functions that mediate host survival to the parasite in *Plasmodium* infections.^5,9^ There is evidence that platelets bind directly to the *Plasmodium*-infected red cells that then secrete platelet factor 4 that builds up within the infected cell and kills the parasite by lysing the food vacuole.^9^ Platelets have a harmful effect in cerebral malaria, contributing to its pathogenesis.^5,10,11^

Schistosomes are parasitic platyhelminths that can cause platelet dysfunction. In mice, platelets secrete various chemokines – such as tegumental apyrase and SmATPDase1 – that can break down the platelet-activating ADP.^12^ Parasites can also produce prostaglandins, specifically prostaglandin D2, which leads to inhibition of platelet aggregation.^13^ Another effect is schistosome tegumental protein Sm22.6-mediated inhibition of thrombin-driven platelet activation.^14^ These protective mechanisms are necessary as platelets are toxic to schistosomes – regardless of prior exposure to schistosomes.^15,16^

Visceral leishmaniasis is commonly associated with anaemia, but other life-threatening haematological complications such as thrombocytopenia and disseminated intravascular coagulation can occur.^17^ Visceral leishmaniasis is an infection of the reticuloendothelial system by the protozoa *Leishmania donovani.* The amastigote form of *L. donovani* proliferates in the spleen, liver and bone marrow. This often leads to massive splenomegaly and splenic sequestration of red blood cells and platelets with resultant thrombocytopenia. A recent study added to the understanding of the indirect effects on platelets by identifying that *L. donovani*-infected mice showed reduced plasma thrombopoietin – likely related to alterations in the liver due to granulomatous inflammation.^18^ Also, bone marrow megakaryocyte cytoplasmic maturation was significantly reduced.^18^ Platelet clearance was increased related to platelet opsonisation and desialylation in the spleen and liver, respectively.^18^

Patients with lymphatic filariasis have dysfunction of their platelets regarding inhibition of aggregation, slower speed of aggregation and increased turnover of platelets.^19^ However, beta-thromboglobulin and soluble P-selectin, related to platelet activation and degranulation, were not elevated in patients with lymphatic filariasis.^19^ This shows a complex modulation of platelet function by the filarial parasites.

Further research is still needed to better understand the role of platelets in hydatid disease. Severe thrombocytopenia and secondary immune thrombocytopenic purpura (ITP) have been described due to hydatid disease.^20^ Some research suggests that certain complement activation may lead to ITP,^21^ and further research has shown complement activating anti-*Echinococcus*-specific antibodies.^22^ There are also data suggesting that hydatid disease may indeed cause platelet dysfunction^8^; the mechanism, however, remains poorly understood.^8^

As shown above, various parasites modulate platelet function through a variety of direct and indirect mechanisms. While data may be lacking regarding the role – or lack of role – that platelets have in the management of hydatid disease, it seems prudent to explore this further. How the *Echinoccocus* parasite evades the innate immune function of platelets remains unclear. Given the above, we postulate that the patient’s subdural haemorrhage resulted from a combination of thrombocytopenia and platelet dysfunction. He had no obvious cerebral echinococcosis or history of trauma or seizures as a possible cause. We further argue that the subdural haemorrhage could be related to possible platelet dysfunction from the patient’s platelets’ response to the hydatid disease, the hydatid disease’s impact on the immune system (and possibly directly on the platelets) and the resultant immune system response that impacted the platelets’ functions. This case report echoes earlier findings and highlights the need for further research into potential immunomodulatory and haematological abnormalities associated with this neglected disease.
